# Kant’s epigenesis: specificity and developmental constraints

**DOI:** 10.1007/s40656-017-0129-2

**Published:** 2017-02-15

**Authors:** Boris Demarest

**Affiliations:** University of Amsterdam, Oude Turfmarkt 143, 1012 GC Amsterdam, The Netherlands

**Keywords:** Kant, Epigenesis, Preformation, Generative force

## Abstract

In this paper, I argue that Kant adopted, throughout his career, a position that is much more akin to classical accounts of epigenesis, although he does reject the more radical forms of epigenesis proposed in his own time, and does make use of preformationist sounding terms. I argue that this is because Kant (1) thinks of what is pre-formed as a species, not an individual or a part of an individual; (2) has no qualm with the idea of a specific, teleological principle or force underlying generation, and conceives of germs and predispositions as specific constraints on such a principle or force. Neither of these conceptions of what is “preformed”, I argue, is in strict opposition to classical epigenesis. I further suggest that Kant’s lingering use of preformationist terminology is due to (1) his belief that this is required to account for the specificity of the specific generative force; (2) his resistance towards the unrestricted plasticity of the generative force in radical epigenesis, which violates species-fixism; and (3) his insistence on the internal, organic basis of developmental plasticity and variation within species. I conclude by suggesting that this terminological and interpretative peculiarity is partly due to a larger shift in the natural philosophical concerns surrounding the debate on epigenesis and preformation. Specifically, it is a sign that the original reasons for resisting epigenesis, namely its use of specific, teleological principles and its commitment to the natural production of biological structure, became less of a concern, whereas unrestricted plasticity and its undermining of fixism became a real issue, thereby also becoming the focal point of the debate.

## Introduction

In the past few decades, Kant’s intervention in the early modern debate on development has been the subject of renewed interest. This is partly because of a general increase in the interest for the life sciences in early modernity and their relation to the development of philosophy. But it is also because Kant uses terms from the embryological theories of his time to describe some of his positions epistemology, his philosophy of history and his philosophical anthropology. Understanding his embryological positions could therefore aid us in understanding his core theories. Unfortunately, even the interpretation of Kant’s position of development is fraught with difficulties.

In several important passages, Kant proclaimed his endorsement of *epigenesis*. This position in the theory of development usually implies that biological individuals and parts are really produced in nature in the processes of reproduction and development. Very often, epigenesis is taken to require a commitment to specific principles or forces that produce, or guide the production of, these new individuals and parts—principles and forces that have a strong teleological component. Finally, the commitment to the real and natural production of new individuals and parts is usually considered to allow for a considerable degree of plasticity in development. In the early 2000s, however, Sloan (2002) and John Zammito (2003) argued that, in spite of Kant’s employment of the *term* epigenesis, his position has many of the traits of the direct rival of this theory, namely preformationism. In fact, so they argue, Kant merely evolved from endorsing an unambiguously preformationist position to a position that was a middle way between epigenesis and preformationism.^1^

In this paper, I will argue that, contrary to what we would expect from his use of typically preformationist terms and concepts, Kant adopted, throughout his career, a position that is much more akin to classical, fixist accounts of epigenesis, although he does reject the more radical, anti-fixist forms of epigenesis proposed in his own time, and does make use of preformationist-sounding terms. I will show that what allowed Kant to do so is a use of the idea of “preformed” and of “germ” that is very different from its use in classical preformationism, which conceives of biological individuals and their complex structures as preformed. First of all, although he seems to reject the classical preformationist idea that biological individuals and their parts are preformed, he accepts that biological species, in contrast, can be thought of as preformed, in the sense of having clear boundaries and being unable to change into different species. Secondly, he shifts from a focus on the preformed “germs” to a focus on the developing force. These two shifts correspond to two important philosophical developments in the eighteenth century. In the first, the concept of “germs” is no longer just used to refer to the fully preformed structure of an individual organism, but is also used to refer to specific laws or reproductive principles for species. A major event in this evolution is Herman Boerhaave’s revival of the Augustinian conception of *rationes seminales* to postulate specific—rather than general—laws for each species.^2^ This view was meant to counteract not the theory that new biological individuals or parts are created in the universe, but rather the view that the developmental forces that produce individuals and parts can also produce new *species*.^3^

The second development is that in which the concept of force acquires more and more respectability due to ideas from Leibnizian and Wolffian metaphysics. In his *German Metaphysics*, Christian Wolff characterized substances primarily by an internal, active force, that actualizes what is contained in them merely potentially (Wolff 1747, pp. 60–68). This view entails that, to the degree that anything is pre-formed here, it may be pre-formed as a predetermined stage in the self-actualization and self-determination of this single force. This conception of the nature of a simple substance as determined solely by intensive properties led to the development, for instance in the work of Du Châtelet (1740, pp. 185–188), of a view according to which nature consists of simple, really indivisible substances without internal figuration, in which the physically and actually indivisible but internally figured and thus possibly divisible “germs” are grounded. This marks a shift where “force” acquires priority over the potentialities that it actualizes, and the internal structure of the organism is seen as “grounded” in the teleological development of the underlying force.^4^

Together, these two shifts lead to a use of “preformation” and “germs” that is no longer primarily concerned with refuting specific productive forces operating in nature, but instead with the limits and constraints to which such forces are subjected. To the extent that Kant remains preformationist, it is because he believes that such forces are not unrestrictedly plastic, even though he is willing to attribute to them a great degree of plasticity (and his talk of germs and predispositions is, somewhat surprisingly, meant to do precisely this). But this “preformationism” is ultimately more a lingering, somewhat mitigated, commitment to species-fixism, according to which whatever produces new biological individuals, cannot also produce new biological kinds—a view that is not so different from that of major fixist epigenesists such as Aristotle or Harvey.

I will defend this interpretation of Kant’s version of “epigenesis” through a detailed analysis of the main passages where Kant discusses theory of development, namely a passage in the *The Only Possible Argument in Support of a Demonstration of the Existence of God* (in Sect. 2), his papers on human races (in Sect. 3), the review of Herder (Sect. 4) and the *Critique of the Power of Judgment* (Sect. 5). I will not discuss the various passages where Kant employs embryological language or metaphors in other contexts, because I believe that judgment on these passages needs to be suspended until we have acquired a clearer picture of the language they employ. In this paper, I will therefore try to show that (1) at no time did Kant endorse a picture that can be understood as classical preformation; (2) Kant only ever attacks views that rival classical epigenesis, such as classical preformationism or metamorphosis; (3) Kant attacked Herder’s epigenesis for its illegitimate expansion of epigenesis, and not for its endorsement of epigenesis; and (4) Kant’s version of epigenesis does allow for both productivity and plasticity. In the course of this paper, I will also address the peculiar shifts in terminology, theoretical concerns and explanatory constraints that could give rise to the peculiarities of Kant’s phrasing and self-characterization.

## The capacity to reproduce

Kant’s first serious discussion of the problem of animal generation occurs in *The Only Possible Argument in Support of a Demonstration of the Existence of God*. This 1763 book consists of two main parts. Relevant here is the second, longer part of the book, where Kant seeks to show how conceiving of all things as metaphysically dependent on God (the topic of the first part) is of use to natural philosophy. He starts off by admitting that design and complexity might be taken to suggest intelligent design, and thus dependence upon God. But he immediately adds that it would be equally a sign of the dependence upon God if all the various complex phenomena could be explained through one single universal principle or law (AA II, pp. 96–97).^5^ Thus, both the irreducibility and the reducibility of phenomena to general natural laws suggest the involvement of God. Passages such as this raise questions regarding the correct interpretation of Kant’s argument. Some (e.g. Tonelli 1959, pp. 43–46) have taken Kant’s position to be radically antiteleological, since it excludes direct design by God, and since Kant himself criticized the anthropocentric conception of teleology in the *Universal Natural History*, which advances a similar position. According to some, there is even a recognizable hint of Spinozism in Kant’s 1755 critique of teleology (cf. Schliesser 2013, p. 426) and his 1763 insistence on God as a unitary, necessitating ground of finite things (cf. Boehm 2014, pp. 31–43). Others, however, insist that Kant’s adherence to teleology is unchanged, but that he changes from an anthropological and design-centered conception of teleology to a non-anthropological one associated with the unity and productivity of nature (e.g. Schönfeld 2000, p. 98; Huneman 2008, p. 93). I believe this final reading makes best sense of the text, and allows us to understand the specific challenge of animal generation better, namely as a difficulty in harmonizing the unity and the productivity of nature:

[I]t would be absurd to regard the initial generation of a plant or animal as a mechanical effect incidentally arising from the universal laws of nature; nonetheless, there is a two-fold question, which has remained unanswered for the reason mentioned. Is each individual member of the plant- and animal-kingdoms directly formed by God, and thus of supernatural origin, with only propagation, that is to say, only the periodic transmission for the purposes of development, being entrusted to a natural law? Or do some individual members of the plant- and animal-kingdoms, although immediately formed by God and thus of divine origin, possess the capacity, which we cannot understand, actually to generate their own kind in accordance with a regular law of nature, and not merely to unfold them? There are difficulties on both sides, and it is perhaps impossible to make out which difficulty is the greatest. (AA II, p. 114)

Kant here contrasts two possible answers to the question of animal generation, and immediately indicates that they are both problematic. According to the first position, which is recognizable as preformationism, animals are not truly generated in nature: they are each individually preformed by God, and only unfold later on. According to the second position, animals have a specific capacity to genuinely produce their offspring, even though this capacity might be unintelligible to us. Kant’s dissatisfaction with either position is, I will argue, that they both violate what he takes to be the central “rule” of natural philosophy, namely

that one must derive a variety of effects from a single cause which is already known, and not immediately suppose the existence of new and diverse operative causes to explain different effects because of some seemingly important dissimilarity between them. Accordingly, it is presumed that there exists a great unity in nature, in respect of the adequacy of a single cause to account for many different kinds of consequences. (AA II, p. 113)

In observing animal generation, there is a tension between our tendency to explain everything in terms of general, preferably mechanical principles, and the seemingly irreducible specificity required for genuine reproduction. In this tension, it seems that we must decide between maintaining our mechanicist generality and losing the capacity to account for generation, or give up mechanicist generality in order to account for the specificity of generation. Kant seems to be convinced that neither option is desirable. I believe that taking this to be Kant’s core issue with animal generation makes better sense of the discussion in the only possible proof, such as this passage:

in the light of everything we know, it is utterly unintelligible to us that a tree should be able, in virtue of an internal mechanical constitution, to form and process its sap in such a way that there should arise in the bud or the seed something containing a tree like itself in miniature, or something from which such a tree could develop. The internal forms proposed by *Buffon*, and the elements of organic matter which, in the opinion of *Maupertuis*, join together as their memories dictate and in accordance with the laws of desire and aversion, are either as incomprehensible as the thing itself, or they are entirely arbitrary inventions. (AA II, p. 115)

First of all, it is important to dispel the misunderstanding that Kant’s criticism of the position advanced by Buffon and Maupertuis is evidence of his rejection of epigenesis, as there are good reasons to believe few authors would recognize this account as epigenesist. In fact, scholars are unsure whether to classify this position as a form of epigenesis or as a form of preformation (cf. Needham 1959, pp. 183–184; Roger 1963, p. 326; Bowler 1971, p. 223; 1973, p. 259). I think it is best to regard it as a third position, reducible to neither epigenesis and preformationism, and a version of the position advanced by Hippocrates (2012, pp. 6–23) and Galen (1916). This position seeks to understand the generation of the animal through the properties and constituents of the elements of the seminal material. Although the Galenic version involved teleology, there are also more mechanicist versions: to a certain extent, Descartes’s intervention into the debate on animal generation was an attempt to show how this process could be explained through mechanical means alone (cf. Hall 1970, p. 64). Epigenesis and preformation, however, are based on the idea that the original position is flawed. Harvey (1847, p. 334), for instance, reintroduced Aristotle’s epigenesis as a theory that downplays the role of material and efficient cause in embryology. Preformation is equally based on the premise that mechanist accounts of animal generation are doomed to fail. Since many preformationists did believe, however, that growth and nutrition could be explained mechanically, they could propose a theory on which organisms are fully preformed and are later unfolded through the perfectly mechanical processes of growth and nutrition. Buffon and Maupertuis revived the older position in the eighteenth century because they believed that, although Cartesian mechanicism could not account for animal generation, a more expanded mechanicism inspired by Newton could. Thus, they argued that principles and forces analogous to gravity (cf. Buffon 1749, pp. 52–53; de Maupertuis 1768, pp. 88–89) could help account for animal generation.

If we ascribe to Kant this view of the general development of embryology in early modernity, we can read the passage quoted above as evidence of Kant’s agreement with the thesis that a mechanicist account of animal generation is doomed to fail, the more recent expanded forms of mechanicism included. I suggest we read his cryptic argument as follows: Buffon and Maupertuis must either maintain that their specific new mechanical principles make animal generation more comprehensible by making it mechanically explainable, or deny that they make it mechanically explainable. On the former option, their position is problematic because it is ad hoc, since mechanicism explains nature in terms of universal, unrestricted principles. Newtonian gravity is admissible as a mechanical effect precisely because it is general in this way, and not an occult quality of a specific subset of objects. The forces and principles introduced by Buffon and Maupertuis, however, account for the specificity in animal generation by themselves being specific. If this is so, however, then these principles do not qualify as mechanical principles. Buffon and Maupertuis can only salvage the mechanicist status of these principles by making them universal. But in such a case they lose precisely the specificity that allows them to account for organic specificity. Hence the dilemma: either this position is mechanicist and fails as an explanation, or it is explanatory but fails to be mechanicist.

Kant is trying to push a similar point in his criticism of preformation:

In this case, the origin of all such organic products is regarded as completely supernatural; it is, nonetheless, supposed that the natural philosophers have been left with something when they are permitted to toy with the problem of the manner of gradual propagation. But consider: the supernatural is not thereby diminished, for whether the supernatural generation occurs at the moment of creation, or whether it takes place gradually, at different times, the degree of the supernatural is no greater in the second case than in it is in the first. The only difference between them relates not to the degree of the immediate divine action but merely to the *when*. As for the natural order of unfolding mentioned above: it is not a rule of the fruitfulness of nature, but a futile method of evading the issue. For not the least degree of the immediate divine action is thereby spared. (AA II, p. 115)

In this passage, Kant contrasts two possible approaches to the idea of supernatural generation. On one version, God intervenes directly in the order of nature to create individual living beings at the moment of their supposed generation. On another version, God has created all individuals at creation and merely allowed them to unfold later on in the history of the world. It seems as if Kant is here contrasting what seems to be a more occasionalist account of animal generation, on which God intervenes at every moment when the otherwise impossible effect would have to occur, with one that is more preformationist because it assumes God only intervened at the creation of the universe itself, and let nature run its course afterward. This is further supported by the fact that preformationists did indeed claim that their theory was superior for decreasing the supernatural amount of intervention required to account for generation. Indeed, Smith (2011, pp. 194–195) has argued that the claims of superiority of preformationism over spontaneous creation were similar to the claims of the superiority of pre-established harmony over occasionalism made by Leibniz. Smith even argues that this accounts for the intimate connection between Leibniz’s theory of causation and his preferences in embryology.^6^ In the passage quoted above, however, Kant claims that this claim of superiority does not withstand scrutiny. I argue here that the reason for this is that the analogy between preformationism and occasionalism on the one hand, and the two listed views on animal generation on the other, breaks down upon closer inspection.

Leibniz has indeed made the claim that occasionalism is inferior to pre-established harmony because it allows for too many miracles, too many interventions of God into the course of nature. The reasoning behind this assumption of superiority is unclear. Antoine Arnauld (G II, p. 84)^7^ took Leibniz to mean that occasionalism requires all causal events to be due to particular volitions of God. But in fact, he remarks occasionalists agree with Leibniz that the occasioning of causes is always or mostly due to general volitions, and not particular interventions. Arnauld thus denies that occasionalism commits one to saying that God intervenes *within time*, that all his interventions are contemporary to the acts that occasion animal generation.

Leibniz responded by stating that the distinction between pre-established harmony and occasionalism is not that the latter is less economical than the former, but that the former preserves a sense in which nature itself is dynamic (G II, pp. 92–93). He advances, against Malebranche and other occasionalists, that the superior position is still the Thomist orthodoxy that God creates and sustains creatures with their causal powers, even though he claims that these causal powers only truly operate within these creatures. Occasionalism robs nature of this autonomous power by stating that creatures do not have causal powers, and that all causal powers reside in God.

This discussion of the differences between occasionalism and pre-established harmony reveals, however, that the analogy between the theories of causation and the theories of generation breaks down. On the occasionalist concept of a miracle, preformationism can decrease the amount of supernatural intervention required by indicating how animal generation could come to pass through a general volition of God, such as a mechanical law of nature. But preformationists precisely assume that animal generation cannot be explained by such general volitions. Hence, they need to assume that each particular individual requires a distinct particular volition to come to be. On Leibniz’s concept of a miracle, preformationism would decrease the amount of supernatural intervention required by indicating how animal generation could come to pass through the proper causal powers of individuals. But again, preformationism maintains that natural entities only have the capacity to develop, not the capacity to reproduce. As a result, the generation of an individual still requires a miraculous intervention.

This allows us to understand Kant’s criticism in the cited passage. Many preformationists seem to have assumed that preformationism is more economical as a theory. Kant notes that this assumption is false: neither on the occasionalist, nor on the pre-established harmony interpretation of a miracle is there any less of a miracle involved in the theory that God created all living beings at the moment of creation than in the theory that God gradually produces new individuals as the history of the universe takes its course. Even worse: preformationism is fatally uneconomical by requiring a distinct principle for each individual. The attempt to salvage mechanicist economy here leads to regarding each biological individual as a stand-alone miracle on a par with that of creation.

These criticisms reveal that the claims of epistemological and metaphysical desirability made by both those in Buffon and Maupertuis’ camp and preformationists are ill-founded. Kant meant to show in this way that a third option, which is recognizable as a form of epigenesis, is preferable: “[t]he purpose of these considerations has simply been to show that one must concede to the things of nature a possibility, greater than that which is commonly conceded, of producing their effects in accordance with universal laws” (AA II, p. 115). This position is less supernaturalist on both notions of a miracle, because it allows for a general volition for each species, and for every individual of this species to have the natural power to propagate. Moreover, this position gives rise, further on in the *Only Possible Argument*, to a rule of Kant’s revised version of physico-theology, which stresses both the unity and the causal autonomy of nature:

One will presume that the necessary unity to be found in nature is greater than strikes the eye. And that presumption will be made not only in the case of inorganic nature but also in the case of organic nature as well. For even in the case of the structure of an animal, it can be assumed that there is a single disposition [*Anlage*], which has the fruitful adaptedness to produce many different advantageous consequences. Initially, we may have supposed that a variety of special provisions must have been necessary to produce such effects. Careful attention to the necessary unity of nature is both consonant with philosophy and advantageous to the physic-theological method of inference. (AA II, p. 126)

Kant thus seems to believe that a theory that attributes a separate principle for whole species is superior to the considered alternatives. Its prima facie disadvantage is that it postulates a separate principle or law for each species. But Kant revealed that the rival positions fare no better: Buffon and Maupertuis end up having to postulate specific powers as well, and the preformationists’ denial of generation forces them to postulate a distinct creative act for each individual, not just for each species. According to Kant, we need to look for sufficiently general natural powers of production, so that no great “variety of special provisions” be necessary to account for the variety of individual and subspecific differences. But, as we see from this passage, some provisions are still necessary, in the form of “dispositions”. In the following section, we will argue that Kant needs such dispositions as constraints on generative force in order to account for its *specificity*.

## The generative force and its constraints

In a series of papers from the 1770s onwards, Kant further discussed his views on animal development in the context of his work on the origin and development of humanity and the different human races. One of these papers is *Of the Different Races of Human Beings*, which was published first as an announcement of his lectures on physical geography in 1775, and again in a revised version as an article in 1777. Its opening is clearly reminiscent of the passages from the *Only Possible Argument* we discussed in the previous section:

The natural division into species and kinds in the animal kingdom is grounded on the common law of propagation, and the unity of the species is nothing other than the unity of the generative power [*zeugenden Kraft*] that is universally valid for a certain manifoldness of animals. […]According to this concept, all human beings on the wide earth belong to one and the same natural species because they consistently beget fertile children with one another, no matter what great differences may otherwise be encountered in their shape. One can adduce only a single natural cause for this unity of the natural species, which unity is tantamount to the unity of the generative power that they have in common. (AA II, pp. 329–330)

Here we find the same call for unity in explanation in nature as in the *Only Possible Argument*: Kant argues that we must understand all human races as being of the same natural species, because it is undesirable to postulate specific further principles merely to account for the minor differences between them. Kant endorses, then, a monogeneticism based on the genealogy of man that could be regarded as a standard creationist story, much as Leibniz did before him (cf. Smith 2011, pp. 269–274). His way of dealing with this monogeneticism, however, cannot be the same as that of Leibniz, because of the rationale lying behind his arguments.

As we saw, in the *Only Possible Argument*, Kant protested against preformation because it had to postulate a distinct creation and a distinct principle for each biological individual. Given Kant’s unwillingness to admit multiple causes to account for multiple human races, I think it is fair to assume he would still have been reticent towards the idea of individual preformation. The main focus of this passage attests to this, namely *the unity of the generative force*. It is important that Kant speaks mostly of a specific generative force, and not of germs and predispositions. The latter come into play only within the framework of a generative force:

The grounds of a determinate unfolding which are lying in the nature of an organic body (plant or animal) are called *germs*¸ if this unfolding concerns particular parts; if however, it concerns only the size or the relation of the parts to one another, then I call them *natural predispositions*. […] As little as chance or physical-mechanical causes can produce an organic body, just as little will they add something to its generative power, i.e., bring about something that propagates itself, if it concerns a special shape or relation of the parts. (AA II, pp. 434–435)

This passage is very important, even if it were only for the misconceptions that it may bring about. Sloan (2002, pp. 233–235) goes through great pains to show that the term “germs” stems unambiguously from classical preformationism, which would explain why Kant believed that “we must consider such occasional unfoldings as *preformed*”. Zammito (2003, p. 83) has concluded from this analysis that “[t]he specific form of *preformation* that Kant endorsed was the sophisticated version developed by Bonnet and Haller in the early 1760s in response to the challenge first of Maupertuis and Buffon and then, more fundamentally, of Caspar Friedrich Wolff”.

However, although the preformationism of Haller and Bonnet was indeed sophisticated, it was no less a preformation. All preformationists believed that environmental and sometimes even hereditary effects could influence the outcome of the process of unfolding. But they also believed that these effects were extraneous to the individual germ itself, which had been preformed at creation. Secondly, they obviously made no distinction between the germ and something like a disposition. In fact, they believed there to be a single germ, which was then unfolded on occasion of incubation and appropriate conception and nutrition. This is certainly not what Kant is talking about here. Finally, Haller and Bonnet thought of such germs as preformed individuals, and therefore Kant’s use of the word “germ” in this context is more akin to that of Boerhaave and Musschenbroek, who considered germs to be specific to whole species, and to be “laws” rather than things.

In line with Boerhaave’s line of thought, Kant’s concern in this passage is more with the unity and constancy of the generative force. He suggests that this unity and constancy is required in order to account for the reproductive and restorative (and probably nutritive) properties of living beings. But he equally wants to account for the adaptive nature of this property, and the inheritability of this adaptedness. As a result, he postulates two kinds of internal constraints on the generative force that determine the outcome of the developmental process. The first kind he calls germs, which he considers to be the factors determining the development of the animal through the generative force. For instance, they determine which parts an organism must have for it to be this kind of organism. The second kind of constraints are not as determinate, but instead determine which possible changes the constraints of the generative force can undergo: they tell us what changes can occur to the sizes, proportions and relations of these parts. The most original aspect of his position is that he believes that not just stabilities, but also changes in form are due to internal constraints rather than outside occasions. This is, in fact, a major shift away from preformation, which suggests that the preformed structure is fully determined, and all real variations are due to outside influences. What is “preformed”, according to Kant, is not a fixed structure, but an internal constraint on the forces and processes of generation and development.^8^

This conclusion is further supported by the context of Kant’s comments, namely the paper’s goal to deliver a better concept of a species:

The school division concerns *classes*, which divide the animals according to *resemblances*, the natural division concerns *lineages*, which divide the animals according to *relationships* in terms of generation. The former provides a school system for memory; the latter provides a natural system for the understanding. The first only aims at bringing creatures under titles; the second aims at bringing them under laws. (AA II, p. 329; translation modified).

As in the *Only Possible Argument*, Kant prefers regarding species as that which is generated through one specific generative force. As we saw, preformationism requires that we understand biological individuals as each created through a special act, and not as the result of the same power, force or activity. Consequently, a species can consist only in the similarities between ultimately unrelated entities. According to Kant, preformation can never have species as natural kinds because it cannot find a principle for the similarities between members of a different species. In absence of such a principle, we can never know which degree of similarity is required to speak of the same species, and we can never know which similarities are required for a specific identity. In absence of this, we will always be forced to classify natural entities on the basis of their surface similarities, realizing that these surface similarities can always be challenged by another choice of criterion.

Kant would therefore regard his own account as superior because it postulates a general principle responsible for specific identity, namely the formative force. This formative force is specified and specifies by means of the germs and dispositions. On the basis of the assumption of such a species identity, we can identify which degree of similarity and which similarities are required to speak of an identity between species. We can also reject the idea that similarities are required to speak of species identity; all that is required is the continuity of production, and no specified amount of dissimilarity can lead one to reject the idea that the same productive force was at work. Finally, as we already saw, Kant’s account entails that the changes in the productive force are internal changes, not external accidents. The productive force is therefore characteristically plastic.

The discussion from the first section can also allow us to understand why Kant nonetheless feels the need to insist on talk of “preformed” elements. We saw that, for Kant, a good account of animal generation must account for the *specificity* of living systems. He rejected preformationism and metamorphosis because they either could not account for this specificity, or gave up on the attempt to give a general, unified, natural account of animal generation altogether. His own account is intended to wed specificity and generality. This becomes clear again in a later paper on the concept of a human race, the 1785 text “Determination of the Concept of a Human Race”. In that text, Kant says of various explanations of differences between human races in terms of various environmental influences that

these and other grounds of explanation would hardly receive credence through the facts adduced to their support, to which one can oppose far better proved ones, if they did not receive their recommendation from an otherwise wholly correct maxim of reason, namely this one: rather to venture everything in surmising from given appearances than to assume special first powers of nature or created predispositions (according to the principle: *principia praeter necessitatem non sun multiplicanda*). (AA VIII, p. 96)

The rationale in this passage is similar to that of the 1763 passages, and suggests that there are good reasons to reject specific principles as explanations for the facts of variation. But, like in 1763, Kant insists that this demand of generality should be balanced with another: “But I am confronted with another maxim which limits the one about doing without dispensible principles, namely, that throughout all of organic nature in all changes of individual creatures their species is preserved unchanged” (AA VIII, pp. 96–97). Kant means two things by this principle: (1) the explanatory demand that the specificity of a species is taken into account; (2) the assumption, elevated to a principle, of the fixity of species. In the remainder of the text, the germs and predispositions are meant to ground a view according to which variation can occur through a developmental process, but without giving rise to the production of new biological kinds. Here too, we see Kant’s puzzling shift between talk of a specific generative force and germs and predispositions lying in the species (rather than individuals). I suggest we interpret these shifts and interchangeability of terminology by attributing to Kant the view that, in order to be sufficiently specific, the generative force must be subject to species constraints, namely germs and predispositions. In the next section, I will argue that Kant comes very close to formulating this exact claim for exactly these reasons in his review of Herder’s *Ideen*.

## The critique of Herder’s unbound epigenesis

In the same year Kant wrote his 1785 paper on human races, Kant reviewed the first volume of Johann Gottfried Herder’s *Ideen zur Philosophie der Geschichte der Menschheit*, which had appeared the year before. In that work, Herder had argued that “Nature, in the infinite variety that she loves, seems to have formed all life on our earth according to one main plasma” (Herder 1869, p. 49; my translation). The idea is that rather than a multitude of specific generative forces, there is only one universal generative force that specifies itself differently in the course of history, thereby giving rise to the variety of species. Kant’s objections to this idea have been taken as a sign of theoretical conservatism and an uncritical commitment to fixism.

Zammito (2003) has made much of Kant’s resistance towards these ideas, and interpreted it as a resistance towards epigenesis, and a predilection for an intermediate position between preformation and epigenesis.^9^ But it is not so clear from Kant’s actual objections in his 1785 review of the first two parts of Herder’s work that his problem with Herder’s position is that it is epigenesist. Commenting on Herder’s conception of a genetic force, Kant writes:

He [Herder] wants to dismiss on the one side the system of evolution [i.e. preformationism] and yet also on the other side the mere mechanical influences of external causes as providing unworkable grounds of elucidation, and he assumes as its cause a principle of life, which appropriately modifies itself internally in accordance with differences of the external circumstances; with this the reviewer fully concurs, only with this reservation, that if the cause organizing itself *from within* were limited by its nature only perhaps to a certain number and degree of differences in the formation of a creature (so that after the institution of which it were not further free to form yet another type under altered circumstances), then one could call this natural vocation of the forming nature also “germs” [*Keime*] or “original predispositions” [*ursprüngliche Anlagen*], without thereby regarding the former as primordially implanted (as in the system of evolution), but merely as limitations, not further explicable, of a self-forming faculty, which latter we can just as little explain or make comprehensible. (AA VIII, pp. 62–63)

Mind that Kant objects not to the idea of a genetic force, which he assumes as well, nor to the idea that it is plastic and can modify itself. The distinction only comes in once this genetic force needs to be qualified. Herder attributes to it an almost unlimited plasticity, which allows it to give rise to all kinds of structures over time. According to Kant, however, the generative force must be internally constrained in order to be explanatory at all. It is in this context that he revisits the preformationist-sounding terminology of the 1770s and the early 1780s, interpreting the “germs” “predispositions” “merely as limitations, not further explicable, of a self-forming faculty, which latter we can just as little explain or make comprehensible”.

This interpretation of germs as the self-limitation of the formative force has also been recognized by Huneman (2008, pp. 208–209), who rightly notes that Kant would have resisted Herder’s account because it invokes an unrestricted transformation of species, which makes species-lineages incomprehensible. But Kant’s criticism goes beyond the insistence on species-fixism. The problem with Herder’s account is also that it does not allow for the specificity of species. According to Kant, I suggest, there must be some limitations on the formative force that explain why offspring typically exhibit important structural similarities with their parents, and why they have the kind of highly specific structures typical of living entities. After all, it is this specificity that an account of animal generation must explain. Like Herder, Kant wants an account that allows for the real reproduction of individuals. However, he still wants this real reproduction to produce specific structures. This is why he takes recourse to a specific and specifying formative force.

## The critique of the power of judgment and generic preformationism

In §81 of the *Critique of the Power of Judgment*, Kant tackles the teleological principle as it is applied to the case of generation. He discerns two distinct ways of accounting for this principle:

If the teleological principle of the generation of these beings (i.e. natural purposes) is assumed (as cannot but be the case), then the cause of their internally purposive form can be grounded in either *occasionalism* or *prestabilism*. According to the former, the supreme world-cause, in accordance with its idea, would immediately provide the organic formation to the matter commingling in every impregnation; according to the latter, it would only have placed in the initial products of its wisdom the predisposition by means of which an organic being produces more of its kind and constantly preserves the species itself, in which a nature that continuously works at their destruction simultaneously makes good the loss of the individuals. (AA V, p. 422)

This passage echoes the *Only Possible Argument*, where Kant, after dismissing mechanical accounts of generation, suggests two other accounts, one of which is occasionalism. The other, however, seems to be that of pre-established harmony. Kant understandably immediately rejects the occasionalist account (AA V, p. 422), because it would make generation fully miraculous and unnatural. This leaves him with what he calls prestabilism:

Now *prestabilism* can in turn proceed in two ways. Namely, it considers each organic being generated from its own kind as either the *educt* or the *product* of the latter. The system of generatings as mere educts is called that of *individual preformation* or the *theory of evolution*; the system of generatings [Zeugungen] is called the system of *epigenesis*. The latter can also be called the system of *generic preformation*, since the productive capacity of the progenitor is still preformed in accordance with the internally purposive predispositions that were imparted to its stock, and thus the specific form was preformed *virtualiter*. Given this, the opposing theory of individual preformation might better be called the *theory of involution* (or that of encapsulation). (AA V, pp. 422–423)

Prestabilism thus comes in two forms: preformation and epigenesis. It may seem peculiar that Kant calls epigenesis generic preformation. But this is less peculiar if we understand Kant to mean that what is “preformed” is the species, not the individual, and that the species is preformed through certain specific constraints. The preformation of the species, Kant says, is virtual rather than real, because it is the guiding idea of the whole which pre-exists the parts of that whole.

It is within the context of such a position that Kant praises Blumenbach both for insisting that all organization comes from organized matter, and for assigning a crucial role to a guiding principle in development (AA V, p. 424). By starting from organization, he takes the original matter of the living being to be not a mere chaotic mixture of elements, nor a homogeneous material, but to be in some way already organized. This organization is not characterized in terms of its resemblance to the later stage of that organization, and therefore not as its preformed version, but rather as the organization giving rise to the latter through a genuinely productive principle: the *Bildungstrieb*. Such an analysis allows us to admit that order does not arise out of chaos, whilst still maintaining that the order that arises does not lie preformed in the order out of which it arises. Nor need we regard this organization as belonging to the generated individual: in order for a living system to be generated, it is required that there already exists another living system which embodies the specific constraints of that species. Moreover, as I have argued, the notions of germs and predispositions are best understood as constraints on the developmental process, and the developmental process as the exercise of the formative force. The formative force, then, acquires its specificity because it is subject to, and incorporates, the specific constraints exemplified by the generating structure (the parent). What pre-exists, again, is the species, not the individual.

So why would we want to read Kant as anything less than a classical epigenesist? The first reason may be that he insists on certain specific constraints on development. However, this would mean that neither Aristotle nor Harvey nor C.F. Wolff, the paragons of epigenesis, were epigenesist, since they all insisted on determinate species characters and specific constraints on development. I believe it is more reasonable to read Kant as criticizing Herder for violating a tenet even classical epigenesists upheld, namely that, although we need not insist on too great a material preformation, we should invoke some determining factors that account for the specificity of organic products.

On the other hand, we may believe Kant to be preformationist because he still modelled these determining factors after the preformationist’s germs. As McLaughlin (2007, p. 287) expresses it: “[Kant’s] germs and predispositions do still fit the determinist eighteenth-century model of pre-given potentialities”. This is true, but it does not seem to be the case that they imply anything more than the eighteenth-century model of determining factors. There is no reason to believe that Kant thought of these potentialities as really preformed and actually prefigured elements. On the contrary, Kant insisted that they are virtually, and not really preformed. Nor is the choice of the term “germ” in any way evidence of an inclination towards preformationism. As already indicated, Boerhaave and some of his students took “germs” to mean precisely the specificity of a certain law to a certain species. Moreover, in 1777, Johannes Nikolaus Tetens, a major source for Kant’s late thought on the topic, remarked that the term “germ is neutral with respect to preformationism or epigenesis, since both theories have to admit of some organization” (Tetens 1777, II, p. 455).

Finally, we may ask why Kant would insist on the pre-given nature of potentialities at all. The reason for this is not only that there need to be factors determining the process of development. We may well want to say that the environment delivers the necessary determining factors. Kant is concerned, however, with explaining why the varieties and adaptations of organisms are not just accidents and deformations due to outside influence: they are equally due to the responsiveness of the internal organic constraints of an organism, organic constraints that can help explain the phenomenon of heritable characters (cf. McLaughlin 2007). In other words, Kant is concerned with showing that an organism is *intrinsically* plastic by including the potential to adapt in its generative force. What is pre-given in an organism, therefore, is its capacity to be epigenetic.

## Conclusion

If what I have tried to show in this paper is right, then Kant’s own version of epigenesis is in fact more like classical epigenesis than like preformationism. And yet, we see both why he clung to preformationist terminology, and why authors such as Sloan and Zammito were right to discern preformationist elements in his theory. Kant seems indifferent to many of the traditional reasons for adopting preformationism, such as reticence towards postulating specific principles in nature, teleology, productive forces and capacities that are difficult to reconcile with sober-minded mechanicism, and the natural production of new individuals and biological structures. In the course of the eighteenth century, however, and under the influence of both continental Newtonians and German Leibnizians, such reticence became less and less sensible. The inquiry into life seemed, in fact, to require some such loosening of the explanatory strictures of classical mechanicism, making specificity and forces fairly unproblematic. In the course of this evolution, the opposition between epigenesis and preformationism transformed as well, as the same issues were no longer at stake. One thing that aided this transformation was the plasticity of the term “germ” itself, a plasticity due to its mixed ancient heritage. After all, it was used to ground a thoroughly plastic notion of nature in the works of the Stoics, to denote the corpuscularian and mechanical first principles of nature in Lucretius’ Epicureanism, and to preserve the pre-planned nature of God’s creation by Christian Neoplatonists such as Augustine. In the mid-eighteenth century, all these different connotations of the term resurfaced to allow for a far greater variety, and far more subtle spectrum, of positions. What may have changed is the variety of positions that could be thought of as somehow preformationist, and this variety included even some classical forms of epigenesis. This also marks a shift in what was regarded as “epigenesis”: not (just) the view that new individuals or biological structures can and do come into existence naturally, but the view that new biological species come into being naturally. Hence, for some, the debate between preformation and epigenesis became primarily a debate on variation, heredity, speciation, and evolution. And it is certainly the case that, in this debate, Kant took a fairly reconciliatory position best described as a mitigated fixism, and, in the terms of the eighteenth century debates in which he engaged, as a mitigated preformationism.

## Footnotes

1 This idea that Kant’s view is in fact a synthesis between two extremes, epigenesis and preformation, can also be found in Pinto-Correia (1997, p. 310). I believe this assessment rests as much on a too extreme view of epigenesis as a too extreme view of preformation.

2 Cf. Boerhaave (1983, pp. 163–169). In the tenth book of *De Genesi ad Litteram libri Duodecim*, Augustine (1983, pp. 399–402) distinguished between two positions that he considered compatible with the thesis that “God created everything at once”, namely the conception on which he created all souls at the moment of creation, and the conception on which he created only the soul of the first man/men, but gave it/them the capacity to engender, according to a formula, new souls. The latter position is an adaptation of the Stoic doctrine of seminal reasons (cf. Knuuttilla 2001, p. 104), and is distinct in that it allows for God to have created everything by creating first individuals and “secret formulae that are causally latent in creation” (Augustine 2002, p. 310). He argues that all biological individuals “carry within them a repetition, so to say, of their very selves, invisible in some hidden power of reproduction” (Augustine 2002, p. 311). Thus, God created everything at once, not by creating or preforming everything individually, but by creating the causal conditions and causal powers that will give rise to them simultaneously.

3 This is most apparent from the way Boerhaave’s student van Musschenbroek (1736, p. 9) uses the passage to refute the concept of “plastic nature”. I would like to thank Pieter Present for pointing this passage out to me.

4 I would like to thank an anonymous reviewer of an earlier draft of this paper for pointing this out to me, and stressing its relevance to my thesis.

5 All references to Kant, with exception of the *Critique of Pure Reason*, are to the pages and volume numbers in Königlichen Preussischen Akademie der Wissenschaften (ed.). *Akademie Ausgabe von Immanuel Kants gesammelten Werken* (29 vols.) Berlin: G. Reimer, abbreviated AA, followed by volume number in Roman numerals. All English translations of passages from Kant are from Guyer, P. and Wood, A. W. (eds.) *Cambridge Edition of the Works of Immanuel Kant*. Cambridge, Cambridge University Press.

6 In offering this view, Smith is suggesting that the link between theories of causation and theories of generation is not an invention of Kant’s, but rather an established element of the early theory. Detlefsen (2003) had of course already taken up Kant’s suggestion of this parallel in order to find out how Malebranche could be both occasionalist and preformationist. Her analysis reveals that there is, after all, no such strong link between theories of causation and theories of generation. But this is not a problem for my view, here, as I do not think Kant himself actually maintained that this parallel is valid; I interpret him as objecting to the typical claim, made explicitly by Leibniz, that certain theories of generation are superior for the same reasons of explanatory economy as certain theories of causation can be said to be superior to others.

7 References to Leibniz’s works and correspondence are from Gerhardt, K. I. (ed.) *Die philosophischen Schriften von Gottfried Wilhelm Leibniz*. Georg Olms Verlag, abbreviated by G, followed by volume number in Roman numerals.

8 Kant’s writings differ greatly in the scope they attribute to these constraints. In his writings on human races, Kant is primarily thinking about ranges of skin color and facial features. In these texts, he also seems to believe no more plasticity is possible. This contrasts with his passages on philosophy of history and anthropology where he leaves the list of possible developments of human intellectual and moral faculties much more open-ended, and suggests that much plasticity still remains. These problems raise further interpretative challenges that go beyond the scope of this paper.

9 He was followed in this by van den Berg (2009: 126–128).
